# A modified ‘skeleton/skin’ strategy for designing CoNiP nanosheets arrayed on graphene foam for on/off switching of NaBH_4_ hydrolysis†

**DOI:** 10.1039/d0ra01892a

**Published:** 2020-07-17

**Authors:** Jinghua Li, Xianyong Hong, Yilong Wang, Yumei Luo, Bin Li, Pengru Huang, Yongjin Zou, Hailiang Chu, Shiyou Zheng, Lixian Sun, Fen Xu, Yong Du, Jianchuan Wang, Federico Rosei, Seifert Hans Jürgen, Ulrich Sven, Xiang Wu

**Affiliations:** School of Material Science & Engineering, Guilin University of Electronic Technology Guilin 541004 PR China sunlx@guet.edu.cn xufen@guet.edu.cn; Guangxi Key Laboratory of Information Materials, Guangxi Collaborative Innovation Center of Structure and Property for New Energy and Materials Guilin 541004 PR China; School of Materials Science and Engineering, University of Shanghai for Science & Technology Shanghai 200093 China; State Key Laboratory of Powder Metallurgy, Central South University Changsha Hunan 410083 China; Institut National de La Recherche Scientifique—Énergie, Matériaux et Télécommunications 1650, Boulevard Lionel-Boulet J3X 1S2 Varennes QC Canada; Karlsruhe Institute of Technology, Institute for Applied Materials Hermann-von-Helmholtz-Platz 1, Bldg. 681 D-76344 Eggenstein-Leopoldshafen Germany; School of Material Science & Engineering, Shenyang University of Technology Shenyang 110870 PR China

## Abstract

CoNiP nanosheet array catalysts were successfully prepared on three-dimensional (3D) graphene foam using hydrothermal synthesis. These catalysts were prepared using 3D Ni–graphene foam (Ni/GF), comprising nickel foam as the ‘skeleton’ and reduced graphene oxide as the ‘skin’. This unique continuous modified ‘skeleton/skin’ structure ensure that the catalysts had a large surface area, excellent conductivity, and sufficient surface functional groups, which promoted *in situ* CoNiP growth, while also optimizing the hydrolysis of sodium borohydride. The nanosheet arrays were fully characterized and showed excellent catalytic performance, as supported by density functional theory calculations. The hydrogen generation rate and activation energy are 6681.34 mL min^−1^ g^−1^ and 31.2 kJ mol^−1^, respectively, outperforming most reported cobalt-based catalysts and other precious metal catalysts. Furthermore, the stability of mockstrawberry-like CoNiP catalyst was investigated, with 74.9% of the initial hydrogen generation rate remaining after 15 cycles. The catalytic properties, durability, and stability of the catalyst were better than those of other catalysts reported previously.

## Abstract 2

Schematic diagram of the preparation, cycles and the kinetics of the Ni/GF/CoNiP nanosheet catalyst.

## Graphical abstract

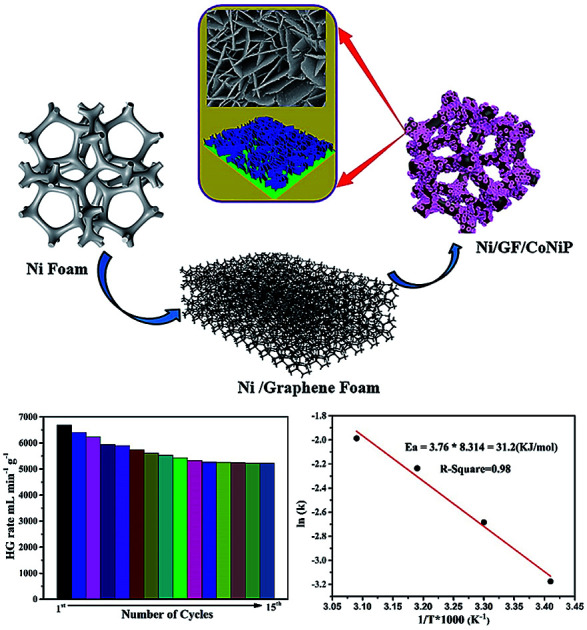

## 1. Introduction

Hydrogen is a promising clean energy carrier and a promising future alternative to fossil fuels.^1–6^ However, the production of hydrogen, and its efficient and safe storage, remain serious challenges that hinder practical applications. Sodium borohydride (NaBH_4_) is a promising system address these issues,^7–9^ owing to its high hydrogen density (10.8 wt%), good storage stability, nontoxicity, and safe reaction conditions. Therefore, NaBH_4_ hydrolysis is considered as an attractive process for producing pure hydrogen at room temperature.^10,11^ However, the catalyst plays a vital role in practical applications, accelerating this hydrolysis reaction in a controlled manner. Among existing catalysts, noble metal catalysts, such as platinum and platinum-based alloys, exhibit the best catalytic activity.^12–15^ However, the high cost and scarcity of such catalysts has limited their widespread use. Therefore, the development of high-performance and low-cost alternative catalysts is a major unresolved challenge.

Transition metals have been reported as new low-cost earth-abundant catalysts that can be further enhanced by doping with P or B,^16^ with examples including Ni–P,^17^ Co–P,^18^ Fe–P,^19^ Co_2_P^20^ and Co–B.^21^ Furthermore, introducing graphene can effectively obtain a higher catalyst surface area. For example, Zhang *et al.*^22^ prepared a cobalt nanocomposite catalyst supported on graphene oxide (GO) that showed excellent catalytic properties for NaBH_4_ hydrolysis. However, π–π interactions and van der Waals forces caused the two-dimensional graphene plate layers to combine, thereby reducing the effective catalyst surface area.^23–25^ To solve this problem, research has focused on 3D graphene. To prepare 3D graphene, nickel foam has been widely used due to its unique structure and low production cost.^16,26,27^ Using nickel foam as a template solves the powder catalyst separation problem, and prevents easy combination and other shortcomings, while allowing convenient collection and recycling. Furthermore, the hydrogen production rate of NaBH_4_ hydrolysis can be controlled by changing the contact area.

**Fig. 1:**
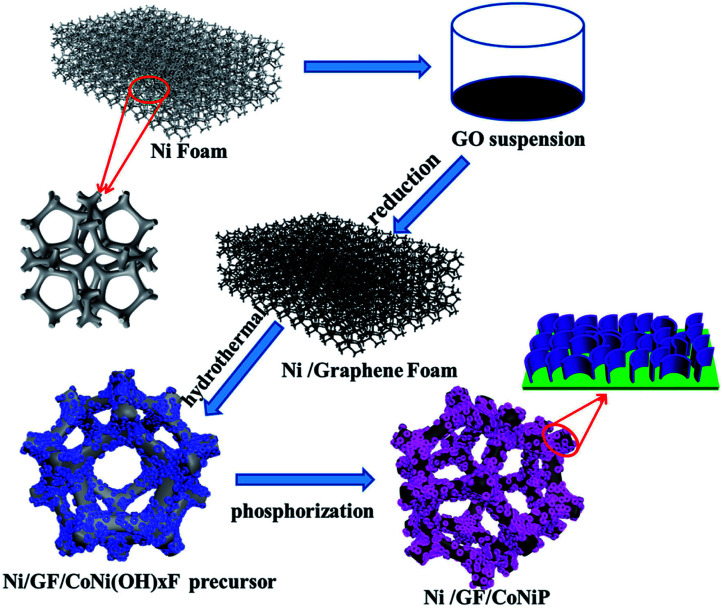
Schematic diagram of the preparation of Ni/GF/CoNiP nanosheets.

Herein, we reported a modified ‘skeleton/skin’ strategy for designing CoNiP nanosheets arrayed on graphene foam for use as on/off switches and reusable catalysts and explored their practical applications as NaBH_4_ hydrolysis catalysts. Fig. 1 shows the preparation procedure of the hydrolysis catalyst based on Ni/GF-supported CoNiP. The whole three-dimensional structure was based on nickel foam, and the graphene foam was prepared using the solution casting method.^27^ The resulting Ni/GF material showed superior performance, good conductivity, a large surface area, and a surface with sufficient functional groups, which not only promoted *in situ* the growth of the CoNiP nanochip array on the Ni/GF bracket, but also helped to improve the hydrogen production rate and circulation stability of NaBH_4_ hydrolysis.

## 2. Materials and methods

### 2.1 Materials

All chemicals were of analytical grade and used without further purification. Sodium borohydride (NaBH_4_), cobalt nitrate hexahydrate (Co(NO_3_)_2_·6H_2_O), nickel nitrate hexahydrate (Ni(NO_3_)_2_·6H_2_O), ammonium fluoride (NH_4_F), urea (CO(NH_2_)_2_), and sodium hypophosphite (NaH_2_PO_2_) were obtained with a purity of 99% from Alfa Aesar Co., Ltd. (Tianjin, China). All experiments were performed using deionized water as solvent.

### 2.2 Synthesis of Ni/GF material

GO was prepared using a modified Hummers method.^28^ Ni foam was used as both the 3D skeleton template and reducing agent to prepare GF. Briefly, a piece of Ni foam (2 × 3 cm) was treated sequentially with acetone and 6 M HCl to remove the oxide layer, then washed with ethanol and deionized water. The Ni foam was then immersed in an aqueous GO suspension (2 mg mL^−1^) at 70 °C for 24 h.^27,29^ During this process, the Ni foam surface was completely covered with the GO solution, and GO was thermally reduced and deposited on the Ni foam to form Ni/GF. The as-obtained Ni/GF was collected and repeatedly rinsed with ethanol.

### 2.3 Synthesis of Ni/GF/CoNi(OH)_*x*_F

The precursor (Ni/GF/(CoNiOH)_*x*_F) was prepared using a hydrothermal method. Co(NO_3_)_2_·6H_2_O (1 mmol), Ni(NO_3_)_2_·6H_2_O (1 mmol), NH_4_F (8 mmol), and urea (10 mmol) were dissolved in deionized water (80 mL) and sonicated for 10 min. Ni/GF was then added to the above solution, which was then transferred into a 100 mL Teflon-lined stainless-steel autoclave, sealed, and heated at 100 °C for 8 h. After allowing to cool to room temperature, the Ni/GF/(CoNiOH)_*x*_F precursor was washed with deionized water and ethanol six times to remove unreacted substances and absorbed Co^2+^ and Ni^2+^ ions. Finally, the precursor was dried at 70 °C for 24 h in a vacuum drying furnace.

### 2.4 Synthesis of Ni/GF/CoNiP

The CoNi(OH)_*x*_F/NF precursor and NaH_2_PO_2_ (0.1 g) were placed in a closed porcelain crucible, with the two substances separated by a carbon cloth (NaH_2_PO_2_ under the cloth and CoNi(OH)_*x*_F/NF precursor above the cloth). The crucible was then heated to 300 °C for 2 h at a heating rate of 3 °C min^−1^ and then cooled to room temperature. The resulting Ni/GF/CoNiP was washed with deionized water and dried at 70 °C in a vacuum drying furnace. Then, the milled powder was taken out and kept in a glove box. For comparison, Co–P, Ni–P, and Co–Ni–P were prepared on blank NiF and Ni/GF using the same method described above. Fig. 1 shows a schematic diagram of the preparation of Ni/GF/CoNiP.

### 2.5 Characterization

The Ni/GF/CoNiP catalyst structures were analyzed by X-ray diffraction (XRD; 1820, Philips, Netherlands). The elemental valence states of the Ni/GF/CoNiP catalysts were determined by X-ray photoelectron spectroscopy (XPS; Thermo Electron ESCALAB 250). The morphologies of the Ni/GF/CoNiP catalysts were determined by scanning electron microscopy (SEM; JSM-6360LV, JEOL Ltd., Japan) and transmission electron microscopy (TEM; JEOL 2010, JEOL, Japan). The Ni/GF/CoNiP catalyst structures were also analyzed using high-angle annular dark-field scanning transmission electron microscopy (HAADF-STEM). The crystalline phase and growth direction of Ni/GF/CoNiP were determined using selected area electron diffraction (SAED). Finally, energy dispersive X-ray (EDX) mapping was used to further analyze the structural composition of the Ni/GF/CoNiP catalyst.

### 2.6 Hydrogen generation measurements

The catalytic activity of Ni/GF/CoNiP in NaBH_4_ hydrolysis was analyzed by testing the hydrogen generation rate. The volume of hydrogen produced was determined from the equivalent displacement of water. The weight of displaced water was recorded with a balance and used to determine the volumetric measurement at constant time intervals. Typically, the Ni/GF/CoNiP catalyst was placed at the bottom of a volumetric flask. At a certain temperature, a solution (10 mL) containing 1.0 wt% NaOH and 1.5 wt% NaBH_4_ was injected into the flask. A water bath was used to keep the system under isothermal conditions. The weight of the CoNiP catalyst was determined according to the following equation:*m*_CoNiP catalyst_ = *m*_Ni/GF/CoNiP_ − *m*_Ni/GF_where *m*_CoNiP catalyst_, *m*_Ni/GF/CoNiP_ and *m*_Ni/GF_ represent the weight of CoNiP catalyst, Ni/GF/CoNiP sample and Ni/GF before hydrothermal treatment, respectively.

### 2.7 DFT calculations

First-principles computations on the basis of DFT were performed using the Vienna *Ab Initio* Simulation Package (VASP).^30^ We use the generalized gradient approximation (GGA)^31^ of Perdew–Burke–Ernzerhof (PBE) to describe the exchange–correlation density. The core–valence interaction was described by the frozen-core projector augmented wave (PAW) method, and the truncation energy for the plane wave expansion wave function was 400 eV. The semicore p states remained fixed for the cobalt pseudopotentials. For the CoNiP@GO structure, we used simplified model calculations, and the unit cell was used to build the metal surface with one layer of graphene for the hydrogen adsorption study. The Brillouin zone was sampled using a 4 × 4 × 1 Monkhorst–Pack mesh. The geometry optimizations were performed by using the conjugated gradient method with the convergence value set at an energy of 10^−4^ eV and 0.05 eV Å^−1^ in force. The vacuum separation was set to more than 20 Å to avoid interactions and periodic images. The effects of van der Waals (vdW)^32^ interactions were considered in the calculations discussed below.

## 3. Results and discussion

### 3.1 Synthesis and characterization

**Fig. 2:**
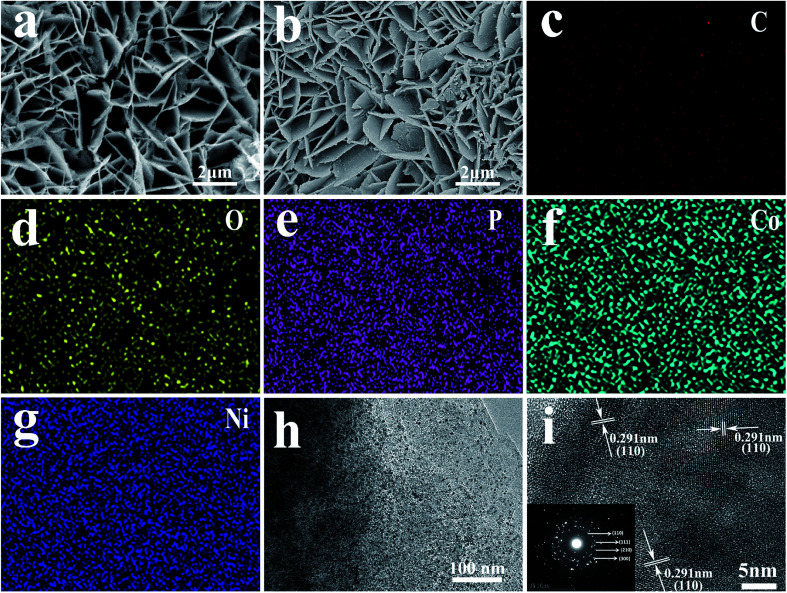
SEM images of (a) CoNi(OH)_*x*_F, (b) Ni/GF/CoNiP nanocomposite; EDX mapping images of (c) C, (d) O, (e) P, (f) Co and (g) Ni. (h) TEM images of the Ni/GF/CoNiP nanocomposite; (i) SAED image of Ni/GF/CoNiP.

Ni/GF was synthesized by solution casting using the Ni foam scaffold as the template,^27^ which provided a large specific surface area for a high active material loading.^33^ The morphologies of NiF and Ni/GF were analyzed by SEM, as shown in Fig. S1a and b.† Fig. S1a† shows a smooth NiF surface, while Fig. S1b† shows a 3D porous structure and rough surface, with a characteristic wrinkle morphology originating from graphene oxide. The reduced graphene oxide nanosheets were assembled on the Ni foam template, forming a ‘skeleton/skin’ structure analogous to that found in the human body, with NiF as the skeleton, and the reduce grapheme oxide coating as the skin.^27^ The structure of Ni/GF was characterized by Raman spectroscopy. As shown in Fig. S2,† the *I*_D_/*I*_G_ values for GO and Ni/GF were 0.97 and 1.23, respectively, which were attributed to the increased sp^2^ domains in the Ni/GF sample, confirming the formation of rGO on NiF. As shown in Fig. S3,† compared with the Ni/GF sample, the Ni/GF/CoNiP nanosheet still retained the unique ‘skeleton/skin’ structure. As shown in Fig. 2a, after hydrothermal treatment, the CoNi(OH)_*x*_F nanosheets uniformly covered the Ni/GF surface. After phosphidation, the as-obtained CoNiP maintained the characteristics of the nanoarray, as shown in Fig. 2b. However, the surface of the phosphidated CoNiP nanosheet was rougher than that of the precursor, and the porous structure was clearly visible. Interestingly, the CoNiP material showed a nanosheet morphology with vertically growth on the Ni/GF. Multiple nanosheets formed a hydrangea-like shape. As shown in Fig. 2c–g, EDX analysis of CoNiP further confirmed the presence of Co, Ni, and P elements in the Ni/GF/CoNiP nanosheet, which was consistent with XPS analysis. TEM imaging shows that CoNi(OH)_*x*_F was deposited on Ni/GF as an ultrathin nanoplate, as shown in Fig. 2h and i. The HRTEM image shown in Fig. 2i contains lattice fringes of 0.291 nm, corresponding to the (110) planes of CoNiP.^29,34^ The SAED pattern (Fig. 2i, inset) corresponds to the (110), (111), (210), and (300) planes.^26,35^ These results confirm the successful preparation of a CoNiP nanoarray on Ni/GF with a modified ‘skeleton/skin’ structure.

**Fig. 3:**
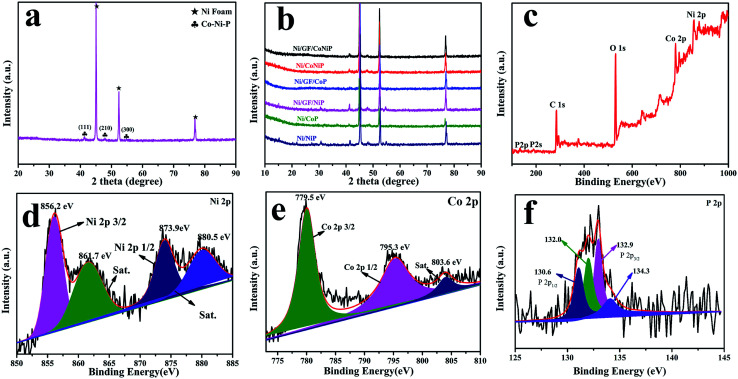
XRD patterns of (a) as-prepared Ni/GF/CoNiP and (b) as-prepared catalysts. (c) Full XPS spectrum of Ni/GF/CoNiP; (d–f) XPS spectra of (d) Ni 2p, (e) Co 2p, and (f) P 2p.

As-synthesized Ni/GF/CoNiP was characterized by powder X-ray diffraction. As shown in Fig. 3a, diffraction peaks at 2*θ* = 44.6°, 52.5°, and 76.4° were assigned to NiF. Furthermore, the phosphidated sample showed peaks at 2*θ* = 41.0°, 47.7°, and 54.4°, which were indexed to the (111), (210), and (300) planes of the CoNiP phase (JCPDS no. 71-2336), respectively.^16,26,35–37^ The XRD results are consistent with HRTEM. As shown in Fig. 3b, no obvious Co–P peaks were found, which might be due to the low loading, good dispersion, and quantum size of Co–P.^37^ Next, the Ni/GF/CoNiP composition was further characterized by XPS.

XPS analysis was used to study the elemental composition and valence states of Ni/GF/CoNiP. The XPS survey spectrum showed the presence of Ni, Co, P, and O elements (Fig. 3c). The presence of oxygen might be due to surface oxidation of CoNiP.^26,38^ High-resolution XPS spectra of Co 2p, Ni 2p, and P 2p are shown in Fig. 3d–f. The binding energy (BE) for Co (779.5 eV) was assigned to Co^2+^ species.^34,39^ The Co 2p XPS spectrum showed two main peaks for Co 2p_3/2_ and Co 2p_1/2_ at 779.5 and 795.3 eV, respectively.^40,41^ The BE for oxidized Ni (856.2 eV) was assigned to Ni^2+^ species. The Ni 2p XPS spectrum showed a doublet for Ni 2p_3/2_ at 856.2 eV and Ni 2p_1/2_ at 873.9 eV.^40^ Furthermore, the Co 2p peak at 803.6 eV and Ni 2p peaks at 861.7 and 880.5 eV were well fitted with three shake-up satellites.^42^ As observed from the P 2p spectrum, two peaks at 130.6 and 132.9 eV are observed, corresponding to P 2p_1/2_ and P 2p_3/2_, respectively, which can be attributed to P bonded to Co and Ni (metal phosphides).^43^ Other peaks located at 132.0 and 134.3 eV can be assigned to oxidized P species,^44,45^ which might be attributed to exposure to air or partial phosphidation of the precursor.^40^

### 3.2 Effect of different types of catalyst on hydrogen generation rate and kinetics of hydrolysis using Ni/GF/CoNiP

**Fig. 4:**
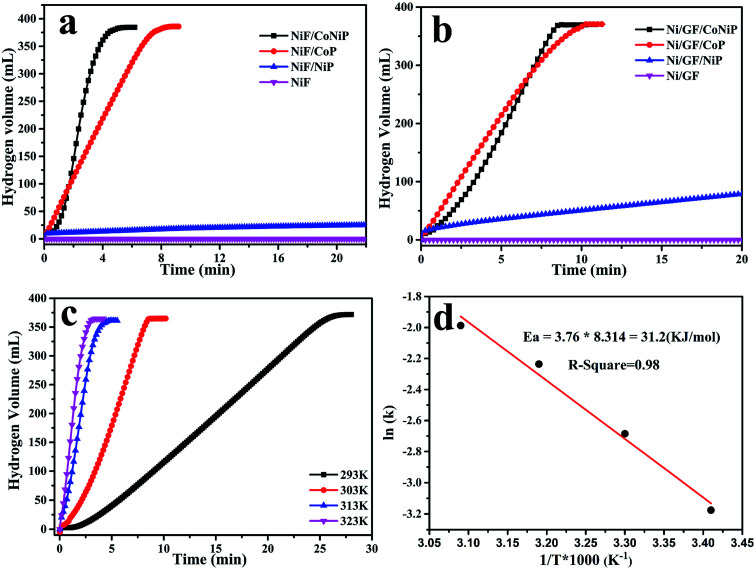
Effect of (a and b) different samples and (c) hydrolysis temperatures on the hydrogen generation rate from NaBH_4_ solution catalyzed by the mockstrawberry-like Ni/GF/CoNiP catalyst using 1.5 wt% NaBH_4_ and 1.0 wt% NaOH solution; (d) the corresponding Arrhenius plot of ln *k versus* 1/*T*.

The catalytic activity of different samples for NaBH_4_ hydrolysis in alkali solution was evaluated. The catalysts were used to hydrolyze a solution (10 mL) containing 1.0 wt% NaOH and 1.5 wt% NaBH_4_ solution. When using only nickel foam used as skeleton structure, the relationship between hydrogen volume produced by NaBH_4_ hydrolysis and reaction time when catalyzed by NiF/CoNiP, NiF/CoP, NiF/NiP, and NiF yields the curves shown in Fig. 4a. NiF/CoNiP had the best catalytic activity. Next, the graphene was poured onto the nickel foam to form the unique modified ‘skeleton/skin’ structure. As shown in Fig. 4b, when NaBH_4_ came into contact with the catalyst, Ni/GF did not release any hydrogen, indicating that it had no catalytic effect. All other catalysts except Ni/GF/NiP afforded 100% of the theoretical hydrogen yield. The loading of Ni/GF/CoNiP, Ni/GF/CoP, Ni/GF/NiP, NiF/CoNiP, NiF/CoP, NiF/NiP were 0.023 g, 0.033 g, 0.061 g, 0.081 g, 0.079 g, 0.071 g, respectively. The HG rates clearly decreased in following order: Ni/GF/CoNiP > Ni/GF/CoP > Ni/GF/NiP > NiF/CoNiP > NiF/CoP > NiF/NiP. The Ni/GF/CoNiP catalyst showed the highest hydrogen generation rate (HGR) of 2289.83 mL min^−1^ g^−1^ at 303 K, which was about twice that obtained using NiF/CoNiP (1620.57 mL min^−1^ g^−1^) and much higher than those obtained using NiF/CoP (683.72 mL min^−1^ g^−1^), NiF/NiP (10.35 mL min^−1^ g^−1^), Ni/GF/NiP (47.28 mL min^−1^ g^−1^), and Ni/GF/CoP (1294.65 mL min^−1^ g^−1^) as catalysts. These results showed that the synergistic interaction among Co, Ni, and P might play an important role in the hydrolysis of NaBH_4_. Furthermore, nickel foam as a skeleton and graphene as a skin provided a high number of active sites for Co–Ni–P catalysts. These active sites might also play a significant role in the enhanced catalytic activity of the Ni/GF/CoNiP catalyst in NaBH_4_ hydrolysis.

To determine the activation energy (*E*_a_) of NaBH_4_ hydrolysis catalyzed by Ni/GF/CoNiP, we performed hydrolysis tests at temperatures ranging from 298 K to 323 K, with all other parameters remaining unchanged. The hydrogen generation yield *versus* reaction time for the hydrolysis of NaBH_4_ in alkaline solution catalyzed by Ni/GF/CoNiP at different temperatures is shown in Fig. 4c. As expected, higher reaction temperatures resulted in an increased HGR. The *E*_a_ can be estimated using the Arrhenius equation:
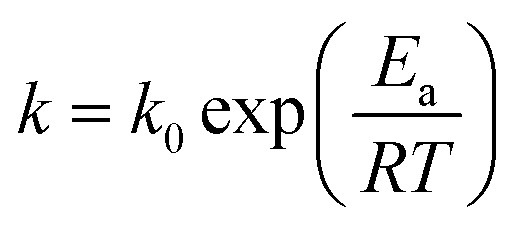
where *k* is the rate constant at various temperatures, *k*_0_ is the pre-exponential factor, *R* is the gas constant, and *T* is the absolute temperature of the hydrolytic reaction. An Arrhenius plot is shown in Fig. 4d. Based on the Arrhenius slope, the *E*_a_ for the hydrolysis of NaBH_4_ catalyzed by Ni/GF/CoNiP was determined to be approximately 33.5 kJ mol^−1^, which was low compared with those of most previously reported catalysts (Table 1). This low *E*_a_ might be due to the synergy among Co, Ni, and P. The CoNiP nanoarray was small and could be evenly fixed to the Ni/GF carrier, which prevented it from accumulating during hydrolysis. Meanwhile, the high-ratio surface area and highly hydrophilic rGO provided a good platform and more active sites for the CoNiP nanoarray. These results indicated that Ni/GF/CoNiP showed excellent catalytic performance for hydrogen generation from NaBH_4_.

**Table 1:** Catalyst systems, synthetic method, HGR, and *E*_a_ values for NaBH_4_ hydrolysis catalyzed by catalysts previously reported in the literature

Catalyst	Synthetic method	*E*_a_ (kJ mol^−1^)	Ref.
CoP/Cu foam	Electroless plating	46.8	46
CoNiMoP/Al_2_O_3_	Electroless deposition	52.4	47
CoNiP/Pd–TiO_2_	Electroless deposition	57.0	48
NiCoP NA/Ti	Electroless deposition	52.68	49
NiCoB	Chemical reduction	62	50
CoNiP/Cu sheet	Electroless plating	53.5	51
CoP/Cu sheet	Electroless plating	60.2	52
CoNiPB	Chemical reduction	29	53
Ni/GF/CoNiP	Hydrothermal	31.2	This work

### 3.3 Stability of Ni/GF/CoNiP

**Fig. 5:**
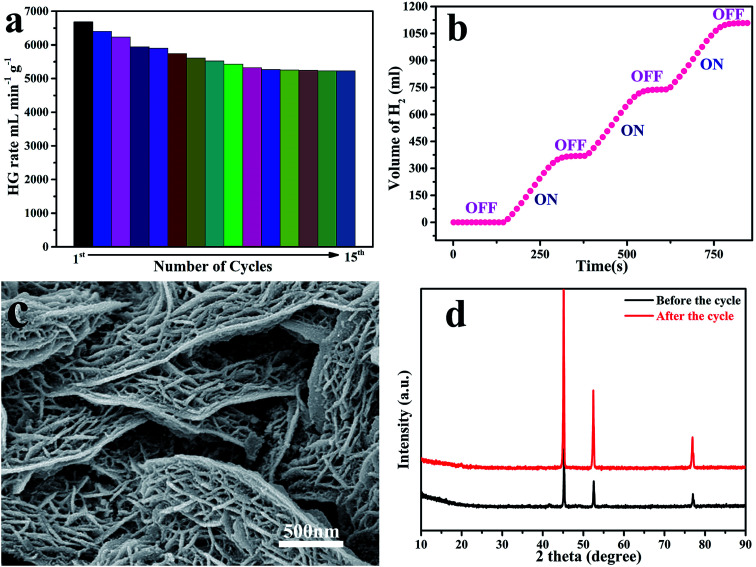
(a) Histogram of hydrogen generation rate *versus* number of cycles for Ni/GF/CoNiP catalyst after 15 cycle tests using 1.5 wt% NaBH_4_ and 1.0 wt% NaOH solution; (b) ‘on–off’ control of H_2_ production. (c) SEM images of the Ni/GF/CoNiP catalyst after 15 cycles; (d) XRD patterns of the Ni/GF/CoNiP catalyst before cycling and after 15 cycles.

Catalyst durability and recyclability are important factors that determine their potential practical applications. Therefore, hydrolysis experiments were performed to recycle the catalyst 15 times at 323 K. As shown in Fig. 5a, Ni/GF/CoNiP had decreased activity in the fourth cycle, but a fairly uniform initial rate increase in each cycle with reasonable catalytic effects. This reduced performance may be caused by the slight loss of catalyst material with each recycling application and a decrease in active sites^54^ However, Ni/GF/CoNiP still retained 74.8% of its initial catalytic activity for NaBH_4_ hydrolysis after 15 recover/reuse cycles. This excellent cycle performance might result from the catalyst having a ‘skeleton/skin’ structure, even though the lamellar structure collapses after 15 cycles, since the nanosheet structure is retained. This phenomenon may decrease the catalyst surface area or number of active sites, resulting in a reduced HGR from NaBH_4_. Fig. 5c shows SEM images of the Ni/GF/CoNiP catalyst after 15 cycles. However, the XRD patterns before and after catalyst cycling were almost identical, as shown in Fig. 5d, and the hydrogen conversion rate was 100%, demonstrating a higher stability and recyclability performance compared with other catalysts reported in the literature (see Table S1†).

The recovery process of our bulk catalysts was much simpler than that of powdered catalysts. As Ni/GF/Co–Ni–P is easily separated from the NaBH_4_ solution, it can be used as an ‘on/off’ switch for on-demand hydrogen production. Fig. 5b shows time control of hydrogen generation with repeated reactivation of the system without compromising catalytic activity. Hydrogen generation on demand can be easily achieved by immersing Ni/GF/CoNiP in solution or removing it from the same solution.^55,56^ The removal of Ni/GF/CoNiP terminates the hydrolysis reaction without releasing hydrogen (‘off’), but hydrogen evolution occurs again after reimmersing Ni/GF/CoNiP in the fuel solution (‘on’).

### 3.4 DFT calculations

DFT calculations were conducted to provide further insight into the promoting effects of the CoNiP@graphene interface on NaBH_4_ hydrolysis. The surface of graphene oxide is complicated. Our calculations implify the model and retain the main feature of the experimental situation. It is comparable between CoP, NIP, CoNiP, CoNiP@graphene. The theoretical hydrogen amount released by NaBH_4_ hydrolysis was 10.8 wt%, with half of the hydrogen atoms provided by H_2_O. The HGR was proportional to the pH value of the BH_4_^−^ solution. NaBH_4_ in aqueous alkaline solution produces hydrogen gas by contact with selected catalyst CoNiP@graphene. The total reaction can be described as follows:^57^

Kaufman and Sen reported that dissociative adsorption of BH_4_^−^ ion would occur on catalyst M (M = Co, Ni).^52^ The NaBH_4_ dehydrogenation process contains four key steps, as follows:

1 2M + BH_4_^−^ ⇔ MBH_3_^−^ + M–H*

2 MBH_3_^−^ ⇔ BH_3_ + M + e_M_^−^

3 M + e_M_^−^ + H′_2_O → M–H′* + OH′^−^

**Fig. 6:**
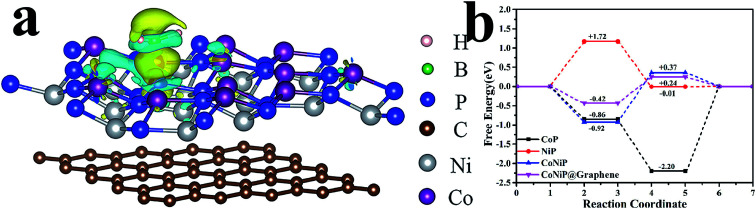
(a) Differential charge map and (b) Gibbs free energy of CoNiP@graphene.

4 M–H* + M–H′* → 2M + HH′

where M–H* represents an intermediate in which a hydrogen atom is adsorbed on an active site on the catalyst surface. In order to distinguish hydrogen atoms from different pathways, we use the H′ symbol to indicate hydrogen atoms from water. Eqn (1) shows that protons from BH_4_^−^ combine with electrons to form adsorbed H atoms. Eqn (3) shows that the active site on the catalyst combines with H in water to form an adsorbed hydrogen atom. As shown in Fig. 6a, the differential charge map clearly indicates the transfer of charge. An analysis of the electronic potential showed that electron transfer occurred from BH_4_^−^ to the CoNiP@graphene surface according to charge accumulation (yellow) and depletion regions (blue). The metallic catalyst would then transfer electrons (e_M_^−^) to molecular H_2_O to generate hydrogen with another active site.

The Gibbs free energy of hydrogen adsorption (Δ*G*_H_) on the active sites can be used as an indicator of hydrogen production activity. The free energy for hydrogen adsorption (Δ*G*_H_) was calculated using the equation Δ*G*_H_ = Δ*E*_H_ + Δ*E*_ZPE_ − *T*Δ*S*_H_, where Δ*E*_H_ is the hydrogen chemisorption energy, and Δ*S*_H_ and Δ*E*_ZPE_ are the differences in entropy and zero-point energy between the adsorbed H and gas phase H_2_. The optimal activity should be obtained at Δ*G*_H*_ ≈ 0.^53^ Excessively high and low Δ*G*_H_ values lead to a decrease in hydrogen production reactivity. As shown in Fig. 6b, the Gibbs free energy of CoNiP@ graphene was closer to 0 than those of CoP, NiP, and CoNiP catalysts. Higher Δ*G*_H_ values suggest weak bonds between the protons and active sites, while lower Δ*G*_H_ values indicate a large surface adsorption energy for hydrogen atoms, both of which hinder the reaction. Compared with CoNiP, the free energy of CoNiP@graphene after graphene adsorption was greatly reduced, owing to the increase in electron density on the metal catalysts near the graphene substrate. Our DFT calculations found that graphene loaded on the CoNiP catalyst showed enhanced hydrolysis activity, which was consistent with the experimental results.

## 4. Conclusions

In summary, NiCoP nanosheet arrays supported on 3D graphene foam with a unique continuous modified ‘skeleton/skin’ structure by hydrothermal method and successfully used as efficient catalyst for the hydrolysis of alkaline NaBH_4_ solution. The resulting Ni/GF/CoNiP catalyst exhibited excellent performance, providing a maximum HGR of 6681.34 mL min^−1^ g^−1^ at 323 K and a low activation energy of 31.2 kJ mol^−1^, along with controllability and durability. Furthermore, thanks to their unique continuous modified ‘skeleton/skin’ reticulate architecture, about 74.9% of the initial hydrogen generation rate was retained after 15 cycles, suggesting excellent cycling stability. As expected, this catalyst was superior to previously reported CoNiP, Co_*x*_P, and NiP catalysts. As the Ni/GF/CoNiP catalyst for activating and accelerating the hydrolysis of alkaline NaBH_4_ was prepared in a simple, cost-effective, and efficient manner, this material has great potential for meeting future demands of hydrogen for fuel-cell-powered vehicles.

## Conflicts of interest

There are no conflicts of interest to declare.

## Supplementary Material

RA-010-D0RA01892A-s001
