# Supplementary material for: A modified ‘skeleton/skin’ strategy for designing CoNiP nanosheets arrayed on graphene foam for on/off switching of NaBH4 hydrolysis
Source: RSC Adv. 2020 Jul 17;10(45):26834–42. doi: 10.1039/d0ra01892a (PMC9055546; doi:10.1039/d0ra01892a)
Supplement: RA-010-D0RA01892A-s001 [file RA-010-D0RA01892A-s001.pdf]

Electronic Supplementary Material (ESI) for RSC Advances.
This journal is © The Royal Society of Chemistry 2020

SUPPORTING INFORMATION

A "skeleton/skin" strategy for designing CoNiP nanosheets arrayed on graphene foam for on/off switching of $NaBH_4$ hydrolysis

Jinghua Li[a,b], Xianyong Hong[a,b], Yilong Wang[a,b], Yumei Luo[a,b], Bin Li[a,b], Pengru Huang[a,b], Yongjin Zou[a,b], Hailiang Chu[a,b], Shiyou Zheng[c], Lixian Sun[*a,b],Fen Xu[*a,b], Yong Du[d], Jianchuan Wang[d], Federico Rosei[e], Seifert Hans Jürgen[f], Ulrich Sven[f], Xiang Wu[g]

a. School of Material Science & Engineering, Guilin University of Electronic Technology, Guilin 541004, PR China.

b. Guangxi Key Laboratory of Information Materials and Guangxi Collaborative Innovation Center of Structure and Property for New Energy and Materials, Guilin 541004, PR China.

c. School of Materials Science and Engineering, University of Shanghai for Science & Technology, Shanghai 200093, China

d. State Key Laboratory of Powder Metallurgy, Central South University, Changsha Hunan, 410083, China.

e. Institut National de La Recherche Scientifique—Énergie, Matériaux et Télécommunications, 1650, Boulevard Lionel-Boulet, J3X 1S2, Varennes, QC, Canada,

f. Trieste University Karlsruhe Institute of Technology, Institute for Applied Materials, Hermann-von-Helmholtz-Platz 1, Bldg. 681, D-76344 Eggenstein-Leopoldshafen, Germany.

g. School of Material Science & Engineering, Shenyang University of Technology, Shenyang 110870, PR China.

*Corresponding authors

Prof. Dr. L. X. Sun

E-mail address: sunlx@guet.edu.cn

Prof. Dr. F. Xu

E-mail address: xufen@guet.edu.cn

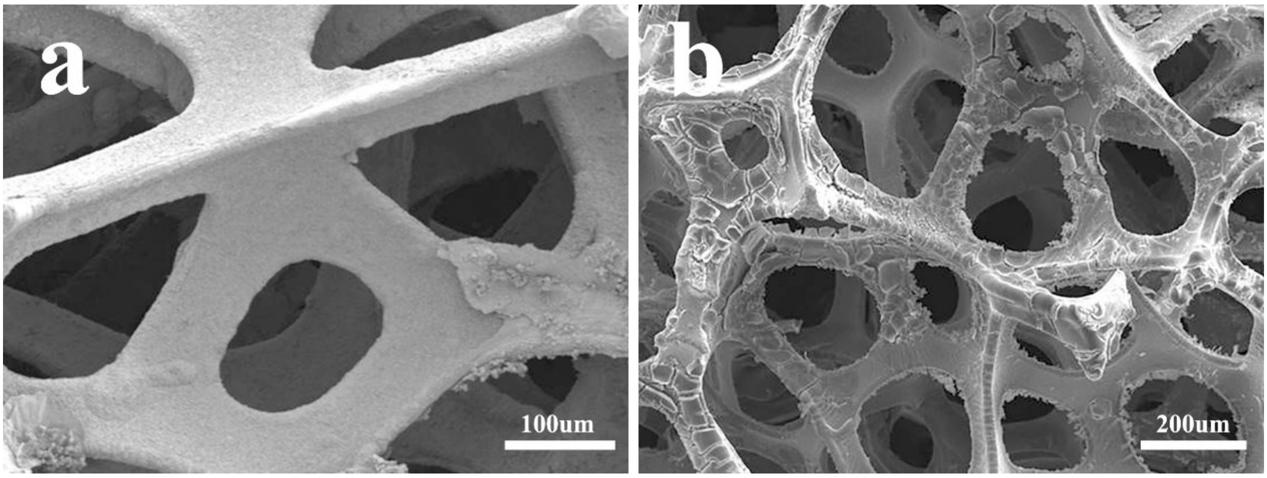

Figure S1. SEM images of (a) NiF and (b) Ni/GF;

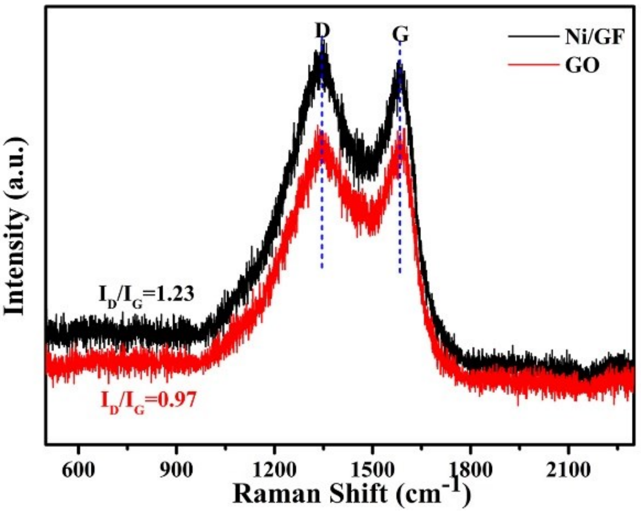

Figure S2. Raman spectra of GO and Ni/GF

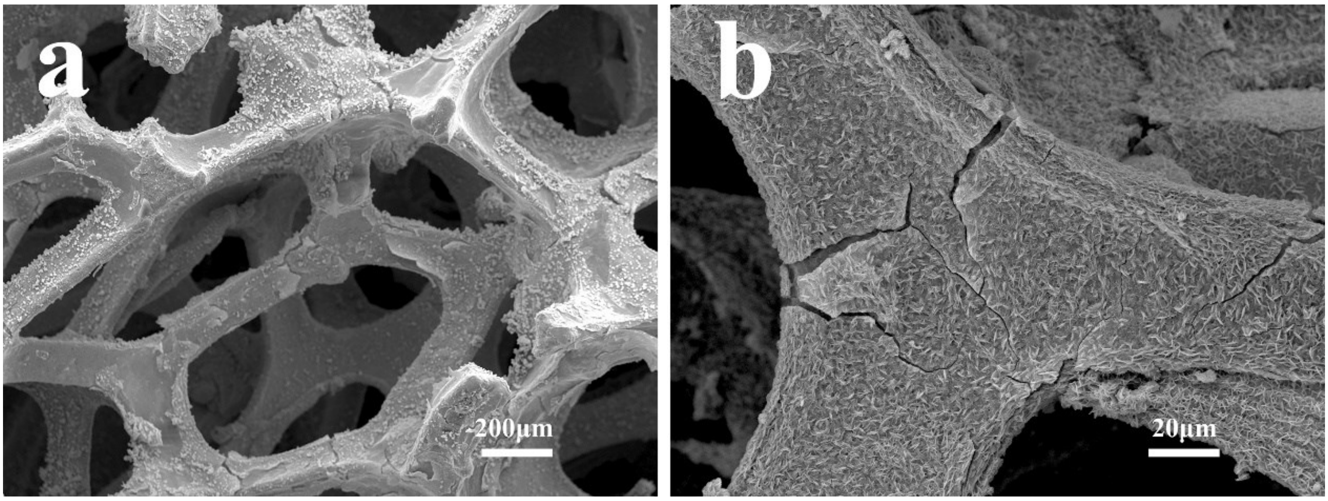

Figure S3. SEM images of Ni/GF/CoNiP

Table S1. Catalyst systems, number of cycles, and cyclic performance for $NaBH_4$ hydrolysis catalyzed by catalysts previously reported in the literature.

Catalyst	Number of cycles	Cycling performance	Ref.
Mn/CeO_2	5	55.0%	1
Co-P	5	67.0%	2
Co-P/CNTs-Ni foam	8	74%	3
$CoCuP/Al_2O_3$	6	66.0%	4
Co/Fe_3O_4-CNTs	8	65.0%	5
Co(0) nanoclusters	15	31.0%	6
Ni/GF/CoNiP	15	74.9%	This work

References

1. S. Duman and S. Özkar, Ceria supported manganese(0) nanoparticle catalysts for hydrogen generation from the hydrolysis of sodium borohydride, *International Journal of Hydrogen Energy*, 2018, **43**, 15262-15274.
2. Y. Wang, Y. Shen, K. Qi, Z. Cao, K. Zhang and S. Wu, Nanostructured cobalt–phosphorous catalysts for hydrogen generation from hydrolysis of sodium borohydride solution, *Renewable Energy*, 2016, **89**, 285-294.
3. F. Wang, Y. Zhang, Y. Wang, Y. Luo, Y. Chen and H. Zhu, Co-P nanoparticles supported on dandelion-like CNTs-Ni foam composite carrier as a novel catalyst for hydrogen generation from NaBH 4 methanolysis, *International Journal of Hydrogen Energy*, 2018, **43**, 8805-8814.
4. Z. Li, L. Wang, Y. Zhang and G. Xie, Properties of Cu Co P/γ-Al 2 O 3 catalysts for efficient hydrogen generation by hydrolysis of alkaline NaBH 4 solution, *International Journal of Hydrogen Energy*, 2017, **42**, 5749-5757.
5. H. A. Bandal, A. R. Jadhav and H. Kim, Cobalt impregnated magnetite-multiwalled carbon nanotube nanocomposite as magnetically separable efficient catalyst for hydrogen generation by NaBH 4 hydrolysis, *Journal of Alloys and Compounds*, 2017, **699**, 1057-1067.
6. M. Rakap and S. Özkar, Hydrogen generation from the hydrolysis of ammonia-borane using intrazeolite cobalt(0) nanoclusters catalyst, *International Journal of Hydrogen Energy*, 2010, **35**, 3341-3346.
